# Specimen collection for electron microscopy.

**DOI:** 10.3201/eid0506.990626

**Published:** 1999

**Authors:** J A Marshall, M G Catton


were "unknown agents." For the sake of objectivity, we based our assumption on the aggregate of information for known pathogens rather than on "expert opinion." Interestingly, however, the Council of Science and Technology's "expert opinion" of the percentage of diarrheal illness due to foodborne transmission was 35% (1), nearly identical to the figure we developed.

As noted in our article, pathogen-specific multipliers for underreporting are needed for many diseases. For lack of a better model, we assumed that the underreporting of toxin-mediated diseases follows the model of *Salmonella*. The alternative Dr. Hedberg suggests, *Campylobacter*, is also a nontoxin-mediated bacterial infection like *Salmonella*, but one for which the degree of underreporting is less well documented. Extrapolating from outbreak data to the number of sporadic cases does indeed have limitations, which is the reason we used it for only the few diseases for which other surveillance data were not available.

Regarding deaths attributed to unknown agents, prospective studies may show that some of these deaths are in fact caused by known agents. However, this would not necessarily lessen the overall impact of foodborne illness: it would merely shift the number of deaths from the unknown category to the known category. The possibility that some deaths attributed to unknown agents are in fact caused by *Salmonella* and other known pathogens supports our use of data on known pathogens to estimate the frequency of foodborne transmission for unknown agents.

Improved estimates will require expanded research into the etiologic spectrum of undiagnosed illness. In the meantime, documenting the substantial impact of foodborne illness neither devalues current surveillance and prevention efforts nor undermines future efforts to determine the causes and impact of foodborne diseases. Our estimates help define gaps in existing knowledge and provide a more rational basis for public health policy than reliance on decades-old data.

**Paul S. Mead, Laurence Slutsker, Patricia M. Griffin, Robert V. Tauxe**
Centers for Disease Control and Prevention, Atlanta, Georgia, USA

### References

1. Foodborne pathogens: risks and consequences. Ames, (IA): Council of Agricultural Science and Technology; 1994.

### Specimen Collection for Electron Microscopy

**To the Editor:** We read with interest the excellent article "Smallpox: an attack scenario," by Tara O'Toole (1). At a critical point in the scenario, the author states, "The infectious disease specialist takes a swab specimen from the … skin lesions… and requests that it be examined by an experienced technician…. electron microscopy shows an orthopoxvirus consistent with variola." In fact, swab specimens of skin lesions for the detection by electron microscopy of viruses such as pox and herpes viruses are far from ideal; the chances of viral detection would be greatly enhanced if a skin scraping were provided to the electron microscopist.

**J.A. Marshall and M.G. Catton**
Victorian Infectious Diseases Reference Laboratory, North Melbourne, Australia

### References

1. O'Toole T. Smallpox: an attack scenario. Emerg Infect Dis 1999;5:540-6.

